# The Lasting Impact of Social Isolation: Behavioral Insights From Former Pet and Entertainer Chimpanzees in a Sanctuary in Spain

**DOI:** 10.1002/ajp.23715

**Published:** 2024-12-18

**Authors:** Emma Chen, Giulia Pipolo, Dietmar Crailsheim, Juliano Morimoto

**Affiliations:** ^1^ School of Biological Sciences University of Aberdeen Aberdeen UK; ^2^ Research Department Fundación MONA Girona Spain; ^3^ Institute of Mathematics University of Aberdeen Aberdeen UK; ^4^ Programa de Pós‐graduação em Ecologia e Conservação Universidade Federal do Paraná Curitiba Brazil

**Keywords:** animal behavior, early life experience, primates, welfare

## Abstract

Human fascination with chimpanzees has driven extensive research on the species, but also led to exploitation by private owners and entertainment industries. These animals often suffer species‐specific inadequate conditions, which can result in the development and display of abnormal behaviors even after rescue. These behaviors highlight the importance for zoos and sanctuaries to actively prevent worsening the effects of previous inadequate treatment by providing apes with social and stimulating environments that support their recovery. We conducted a 2‐month behavioral survey on two groups of former pet and entertainment chimpanzees (*n* = 10) at the Fundación MONA sanctuary in Spain. From 116 h of instantaneous scan observations, we documented individual abnormal behaviors (e.g., self‐poking, overgrooming, self‐scratching). We assessed the association between the occurrence of these behaviors and individuals' origin, early history, age at rescue, and pre‐rescue social conditions. We found no significant difference in the frequency of abnormal behavior between pet and entertainer chimpanzees, and between individuals born in captivity versus in the wild. Moreover, we observed that the frequency of abnormal behaviors increased with age at rescue for previously isolated individuals, but the correlation disappeared for those socially housed pre‐rescue. These findings suggest that early social isolation and a late age at rescue may impose long‐term changes on chimpanzees' behavior, and they emphasize the importance of accounting for age at rescue and previous housing conditions in care management and rehabilitation procedures.

## Summary

- We studied the long‐term impact of past inadequate treatment and unfavorable living conditions on the behavior of two groups of former pet and entertainment chimpanzees at Fundación MONA.
- We observed no significant differences in abnormal behavior between pet and entertainer chimpanzees, or between captive‐born and wild‐born individuals.
- Social isolation before rescue significantly increased the frequency of abnormal behavior for individuals rescued later in life, whereas for chimpanzees previously housed socially, age at rescue did not impact the frequency of abnormal behavior.

## Graphical Abstract

The figure illustrates the impact of pre‐rescue housing conditions and age at rescue on abnormal behavior in sanctuary chimpanzees. Chimpanzees isolated prior to rescue (yellow data points) show a positive relationship between age at rescue and scans spent displaying abnormal behaviors—rescues occurring later in life are associated with higher percentages of scans displaying these behaviors. In contrast, chimpanzees housed socially pre‐rescue (green data points) do not show this trend, as their age at rescue does not influence the frequency of abnormal behavior.

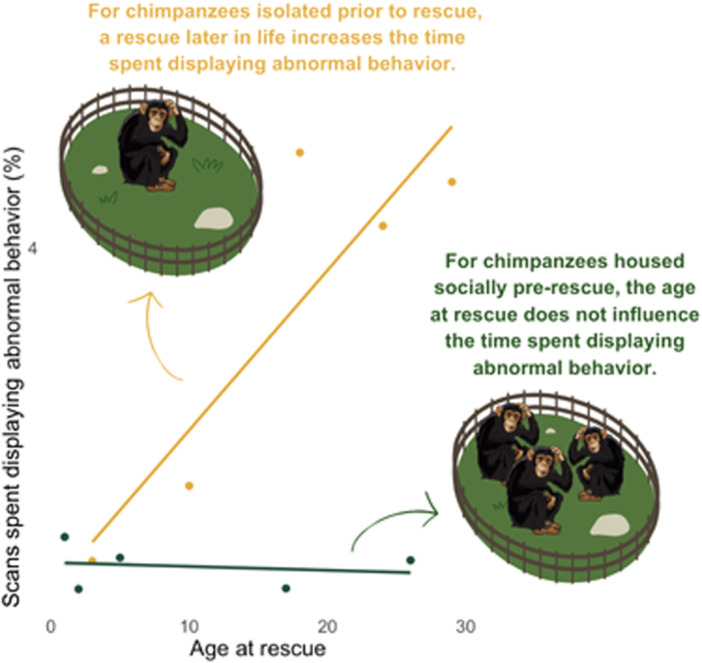

## 1 Introduction

Chimpanzees (*Pan Troglodytes)* have long been subjects of extensive research due to their genetic and phenotypic similarities to humans (Padrell et al. 2020). However, this profound interest in chimpanzees has led to their exploitation in entertainment industries or by private owners. Some European countries have imposed complete (e.g., Scotland, Belgium, Sweden) or partial (Spain) bans on wild animals in circuses, whereas other countries have abstained from any regulation pertaining to animal use for entertainment (e.g., Italy, France) (Eurogroup for Animals 2021). Despite bans on the import of exotic animals, the illegal wildlife trade still thrives, and among apes, the live trade of chimpanzees dominates the market, with an estimated annual global market value from US$1.6 million to US$6.4 million (Arcus Foundation 2021; Feliu et al. 2022). Private owners often acquire these illegally traded chimpanzees as pets, housing them under inadequate conditions, with minimal exposure to other chimpanzees (Freeman and Ross 2014). When the chimpanzees reach maturity, the owners frequently seek to part with them, as they can no longer confine the animals in small cages nor control their behavior (Feliu et al. 2022).

**Figure 1:**
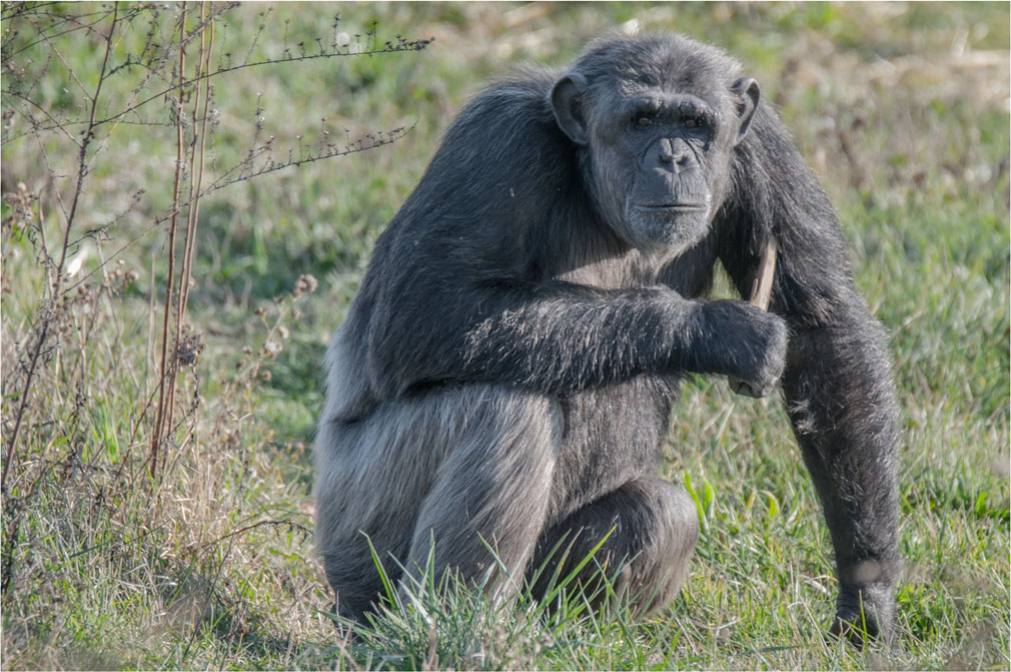
Self‐poking with a stick (an example of abnormal behavior) by Coco, a formerly isolated pet chimpanzee in our study, rescued at 18 years old.

Chimpanzees exploited as pets or entertainers typically endure unfavorable living conditions, including housing in unsuitable environments, temporary or permanent social isolation from conspecifics, and inadequate diets, to name a few. Consequently, they tend to develop and exhibit abnormal behaviors (Figure 1), that is, behaviors that deviate from the norm for wild chimpanzees and may be associated with psychiatric disorders described in humans such as anxiety, depression, and posttraumatic stress disorder (Brüne et al. 2006). Rehabilitation of these individuals is a protracted process, and they likely require long‐term specialized care (Crailsheim et al. 2020; Feliu et al. 2022). Thus, the relocation of chimpanzees to primate sanctuaries provides them with a safe and supportive environment where individuals can live in a social space with conspecifics and receive professional care. In this context, the Fundación MONA (Fundaciónmona.org 2023) is a wildlife sanctuary located in Northern Spain that has been rescuing and rehabilitating chimpanzees and Barbary macaques since 2001. The center is part of the European Alliance of Rescue Centers and Sanctuaries (EARS.org). At MONA, the treatment of primates is guided by four principles, which include (1) the provision of suitable housing, social interaction, and (2) species‐appropriate activities, (3) knowledge of individuals' histories, and (4) minimal human interaction—only arranged visits and veterinary support (Llorente et al. 2015). MONA offers an ideal setting for an observational, noninvasive behavioral investigation that respects the chimpanzees' welfare, as there are few human visitors, and even those visitors were shown to have minimal impact on the animals' behavior. For instance, a study by López‐Álvarez et al. (2019) found that human visits only had a minor effect on the chimpanzees' daily routines, primarily leading to increased human interaction behaviors—91.9% of which were positive or neutral, such as the chimpanzees closely observing visitors.

The impact of past negative events on chimpanzee behavior has been previously investigated, revealing varied outcomes based on different stressors and communities (Brüne et al. 2006; Bloomsmith et al. 2006). Captive‐born chimpanzees may experience an environment with more external stimuli and peers' social interactions, and develop enhanced social skills compared to their wild‐caught counterparts (Davenport, Rogers, and Rumbaugh 1973; Llorente et al. 2015; Crailsheim et al. 2020). However, an early study by Suomi, Harlow and Kimball (1971) on rhesus macaques showed that individuals born and reared in laboratory settings under socially restrictive conditions displayed higher levels of rocking, self‐biting, and self‐clasping compared to individuals caught from the wild within their first 2 years of life. While the specific dynamics of laboratory‐born rhesus macaques raised in partial isolation may differ from those of chimpanzees born and raised in captivity under non‐laboratory conditions, it is important to acknowledge that captivity conditions can sometimes be associated with social deprivation. Therefore, some captive‐born chimpanzees, particularly those raised in inadequate facilities and in isolation, could also experience more social deprivation, lacking access to appropriate peer interactions.

Captive chimpanzees can be housed in a wide range of contexts, often dictated by human use or circumstances. These contexts include zoos, research laboratories, sanctuaries, and private ownership settings, and each context presents unique challenges for the animals' welfare and behavioral development (Bradshaw et al. 2008; Llorente et al. 2015). In the current study, we focus on rescued chimpanzees who were previously housed as pets or entertainers. We categorized the subjects as “entertainment” if they had been primarily involved with circuses and/or commercial agencies; and “pet” if they had been kept in human households for exclusively private purposes (‘entertainment’ animals may also have been housed in the trainer's house during youth).

The distinction between the effects of experience as pet and entertainment chimpanzees remains unclear. Both groups are highly exposed to humans, a factor known to elevate stress levels in captive settings, and reduce behavioral diversity (Freeman and Ross 2014; Jacobson et al. 2017; Kühl et al. 2019). Previous research suggests that performer chimpanzees may exhibit higher behavioral competence correlated to the enriched environments they inhabit and the potential for conspecific housing (Freeman and Ross 2014; Llorente et al. 2015). However, they also might be subjected to a stricter training routine than pet animals, including negative reinforcement, such as training chimpanzees to avoid interactions with whips or sticks to increase the frequency of desired behaviors in a circus show (Iossa, Soulsbury, and Harris 2009).

Although Baker (2000) found no difference in the levels of abnormal behaviors exhibited by old versus younger captive chimpanzees, more recent literature indicates that older captive chimpanzees display more abnormal behaviors than their younger counterparts (Neal et al. 2019b). Moreover, Veenema et al. (1997) recorded a significant increase of stress‐related behaviors with age in long‐tailed macaques housed in captivity. However, these do not represent analyses of the influence of the duration of inadequate treatment experiences on such behaviors in chimpanzees. Reimers, Schwarzenberger and Preuschoft (2007) demonstrated that the duration of deprivation impacts former laboratory chimpanzees' personalities and coping strategies. Indeed, the individuals who were isolated earlier in life and for a longer period showed a higher stress response during rehabilitation, suggesting that former pet and entertainer chimpanzees could show a similar pattern.

Extensive research underscores the importance of a social environment for typical chimpanzee development during infancy, emphasizing the need for a mother figure, whether human or conspecific, in early development (Bloomsmith et al. 2006; Botero, MacDonald, and Miller 2012; Ongman et al. 2013; Maki, Fritz, and England 1993). Particularly, captive chimpanzees who underwent an early impoverished rearing have been shown to develop stereotypic behaviors, including rocking and self‐clasping, and cognitive and social deficits that persisted after many years of environmental enrichment and social group maintenance (Menzel, Davenport, and Rogers 1963; Davenport 1963; Turner, Davenport, and Rogers 1969; Davenport, Rogers, and Rumbaugh 1973). Moreover, early responsive care, supporting the development of species‐typical socio‐emotional and communicative skills, was found to positively impact the emotional and cognitive development of captive chimpanzees (van Ijzendoorn et al. 2009).

Early social deprivation has been directly linked to heightened anxiety levels in chimpanzees (Ortín et al. 2019; Ferdowsian et al. 2011), marmosets (Cinini et al. 2014), and rhesus macaques (Kempes et al. 2008). Additionally, studies demonstrated the advantages of housing larger groups of chimpanzees together to reduce aggression and anxiety‐related behaviors (Neal Webb, Hau, and Schapiro 2019a; Baker and Easley 1996; Baker 2004). Together, this evidence highlights the lasting effects of disrupted social experiences and the critical role of group dynamics in mitigating those effects and promoting well‐being.

The current research aims to add to existing literature on abnormal behavior in chimpanzees by providing a more detailed description of how early life experiences impact the current behaviors of rescued chimpanzees, especially by examining the link between their age at rescue and their social condition pre‐rescue, as well as the explicit comparison of pet and entertainment chimpanzees. Indeed, the comprehensive array of information available on the chimpanzees' past allowed us to perform an examination of the diverse factors contributing to abnormal behavior, whereas previous research often lacked such detailed data about the animals' history.

In this study, we investigated the correlation between chimpanzees' individual history and abnormal behavior observed in the study period, addressing key questions regarding the impact of origin, early history, age at rescue, and social conditions before rescue. Based on the previously presented literature, we predicted that wild‐born chimpanzees would spend more time displaying abnormal behavior than their captive‐born counterparts, whereas former pet chimpanzees were expected to show more abnormal behavior compared to those involved in entertainment. We also predicted that the duration of inappropriate conditions would correlate with abnormal behavior, especially in older captive chimpanzees; and that social rearing would mitigate the display of abnormal behaviors compared to chimpanzees raised in isolation.

Beyond enhancing our understanding of abnormal chimpanzee behavior, this research has broader implications for animal welfare, emphasizing the need to raise awareness about the consequences of exploiting wild primates for entertainment or private ownership. Overall, our study provides valuable insights into how rescued chimpanzees' behavior may be impacted by past inadequate living conditions, highlighting the importance for sanctuaries and zoos to consider each individual's previous history to better support their physical and mental wellbeing post‐rescue.

## 2 Methods

### 2.1 Study Population

Our observational study took place at Fundación MONA, a sanctuary for primates located near Girona, Spain. We collected data on two chimpanzee groups, *Bilinga* and *Mutamba*, which comprised of five individuals (three males and two females) each; and were housed in Continuous Full Contact conditions (group)—more than two animals are housed in one space, enabling complete tactile contact. As illustrated in Figure S1, both the *Mutamba* and *Bilinga* groups have access to two separate outdoor enclosures, labeled Enclosure 1 (2420 m^2^) and Enclosure 2 (3220 m^2^). These enclosures are equipped with various amenities such as climbing ropes, wooden towers, water dispensers, and enrichment devices. For safety, the area is enclosed with electrified wires, and a bamboo wall is in place to ensure the animals' privacy, except for a few designated viewpoints (López‐Álvarez et al. 2019). All chimpanzees in the study were previously exploited by circuses, advertisement companies and/or private owners for entertainment or as pets before being moved to MONA. For this research, we assessed each individual's display of abnormal behaviors based on the variables of the animal's origin (captive or wild), early history (entertainer or pet), age at rescue, and social conditions before rescue (social group or isolated). We did not investigate the impact of current living conditions, as all chimpanzees were observed while living in a social group (both groups composed of three males and two females). A summary of the individual information of each chimpanzee is presented in Table 1. We considered chimpanzees as adults if they were older than 15 years old, and juveniles (comprising infancy, childhood, and adolescence) from birth to 15 years old, based on a categorization used by Goodall in 1983, 1986 and multiple subsequent studies (including Kawanaka 1989; Sandel, Reddy, and Mitani 2017; Sandel, Langergraber, and Mitani 2020). During the data collection phase, the chimpanzees' age ranged approximately from 20 to 41 and their age at rescue from 1 to 29.

**Table 1:** Summary of the main information available for each chimpanzee in our study groups, comprising its group at MONA, its sex, and the info about the individual's previous living conditions.

Group	Chimpanzee name	Sex	Origin	Early history	Age at rescue	Years at MONA	Social conditions before rescue
Mutamba	Africa	Female	Wild	Pet	10	14	Isolated
	Bongo	Male	Captive	Entertainment	2	21	Social
	Juanito	Male	Captive	Pet	1	19	Social
	Marco	Male	Captive	Entertainment	17	22	Social
	Waty	Female	Captive	Entertainment	5	21	Social
Bilinga	Nico	Male	Captive	Pet	3	19	Isolated
	Victor	Male	Captive	Pet	24	17	Isolated
	Cheeta	Female	Wild	Entertainment	29	8	Isolated
	Coco	Female	Wild	Pet	18	11	Isolated
	Tom	Male	Wild	Entertainment	26	12	Social

*Note:* “Origin” indicates if the chimpanzee was born in the wild or in captivity. “Early history” refers to the chimpanzee's commercial or private use before its arrival at MONA, best determined from available records. We categorized the subjects as “entertainment” if they had been primarily involved with circuses and/or commercial agencies; and “pet” if they had been kept in human households for exclusively private purposes. We calculated the “Age at rescue” based on the year of arrival at MONA and the estimated year of birth. The years spent at MONA refer to how long the subjects have been at the sanctuary at the time of data collection. The “Social conditions before rescue” refer to the chimpanzees' primary housing conditions, i.e., isolated or socially housed with conspecifics.

### 2.2 Data Collection

We collected behavioral data between August 1 and September 24, 2023, using instantaneous scan sampling (Altmann 1974) at 2‐min intervals for all individuals of one group during 20‐min observational sessions. For each session, the observers recorded the behavior of five individuals simultaneously every 2 min, obtaining 10 time points that allowed us to calculate the frequency of behaviors, or, more precisely, the percentage of scans displaying the behaviors present in the ethogram. The data collection window was limited to the hours between 10 a.m. and 6 p.m., coinciding with the times when the chimpanzees had access to the naturalistic outdoor enclosures. We gathered data using tablet devices with the *ZooMonitor* behavioral monitoring app, developed by the Lincoln Park Zoo (Ross et al. 2016). Observation sessions were evenly distributed between mornings and afternoons on randomly selected days of the week, from Monday to Sunday. The average number of observation sessions conducted per day was 9 ± 4; thus, we recorded an average of 3 h of observation per day. The observers were stationed on one of the two observation towers to oversee the respective enclosure, and they conducted 1–3 sessions in a row, switched to another group and/or took a break before continuing.

Both the first author (EC) and other observers at the sanctuary received training from the Research Coordinator at MONA and were assessed as official observers after passing a three‐step quality and interobserver reliability test with an agreement rate exceeding 85% with the research staff at MONA. Data collected during training sessions were not included in the analysis. The *ZooMonitor* app records data based on an ethogram established by Fundación MONA, used in multiple studies conducted at the sanctuary (Llorente et al. 2015; Padrell et al. 2020; Feliu et al. 2022, Ayuso et al. 2023) and reviewed periodically, with the last update being in July 2023. The ethogram encompasses various behavior categories, including *solitary behavior*, *interactive behavior*, and other information such as *proximity*, *object use*, and *elevation* level. Importantly, it also collects data on *abnormal behavior (solitary)*, which was used in this study. This included behaviors that are (a) not typically observed in the wild or (b) exhibited excessively or as an inadequate, nonadaptive response to a specific situation (Mason 2006; Lutz et al. 2022, Ayuso et al. 2023). The abnormal behaviors recorded at the sanctuary and included in our research are primarily of auto‐stimulating nature, directed to oneself, and may differ from the ones observed in chimpanzees in other captive contexts. The ethogram collected in the app, including all the solitary abnormal behaviors considered in this study, is given in Table S1.

### 2.3 Data Analysis

We conducted statistical analyses in R version 2023.09.0+463 (R Core Team 2018). For each chimpanzee, we calculated the percentage of scans spent displaying abnormal behavior by dividing the total count of abnormal behavior by the total count of observed behaviors (excluding the ‘not visible’ entries) and multiplying by 100. Using “dplyr” (version 1.1.0; Wickham et al. 2023) we calculated the individual contribution (%) to the total percentage of scans spent displaying abnormal behavior per variable (origin, early history and social conditions before rescue). We used “ggplot2” (version 3.4.1; Wickham 2016) to visualize the percentage of scans spent displaying abnormal behavior against the interaction of age at rescue and social conditions before rescue. We considered age as a continuous variable, and we present two slopes obtained from linear models fitted separately for the individuals that had been raised in isolation and those raised in social groups. To test for the relationship between age at rescue, abnormal behavior, origin, early history and social conditions before rescue, we fitted a linear mixed model (LMM) using the “lme4” package (version 1.1.34; Bates et al. 2015) and “lmerTest” (version 3.1.3; Kuznetsova, Brockhoff, and Christensen 2017). The social group at MONA (*Bilinga/Mutamba*) was used as a random effect, the percentage of scans spent displaying abnormal behavior was the dependent variable, whereas origin, early history, the age at rescue, social conditions before rescue and their interaction were used as predictor variables. We used an analysis of variance (ANOVA) to examine the fixed effects of the model, with the **F‐statistic** being calculated to assess the significance of the model fit when including each of the predictors. The corresponding ***p*‐values** from the ANOVA output show whether each predictor's effect is statistically significant, if *p*‐values were below the 0.05 threshold, indicating that the effect observed is unlikely to have occurred by chance. We tested for the interaction between early history and social conditions before rescue, which was not statistically significant (F_1, 2.1_ = 0.56, *p* = 0.53) and was therefore removed from the final analysis. We checked for multicollinearity issues between the predictor variables using the VIF function in the “car” package (version 3.1.2; Fox and Weisberg 2019). We tested the normality of residuals with a Shapiro‐Wilk normality test.

## 3 Results

### 3.1 No Differences in Abnormal Behavior Between Captive‐Born and Wild‐Born Individuals, and Between Pet and Entertainment Chimpanzees

A total of 116 h of behavioral data were analyzed for abnormal behaviors. We observed that chimpanzees spent 1.73 ± 2.09% of their time displaying abnormal behaviors. Of the 10 individuals studied, 6 displayed abnormal behavior in less than 1% of scans (Table 2), leaving the remaining four as the primary contributors to the observed abnormal behavior. Notably, Coco, Cheeta, and Victor (all previously isolated and rescued in adulthood) accounted for a substantial share, contributing 29.4%, 27.5%, and 24.5% of the total abnormal behavior, respectively.

**Table 2:** Percentage of scans spent displaying each abnormal behavior identified per individual.

Chimpanzee name	Scans spent displaying abnormal behavior (%)	Coprophagy (% of the individual abnormal behavior)	Overgrooming (% of the individual abnormal behavior)	Rocking (% of the individual abnormal behavior)	Self‐poke (% of the individual abnormal behavior)	Self‐scratch (% of the individual abnormal behavior)	Touch forehead (% of the individual abnormal behavior)
Africa	1.25		100				
Bongo	0.06	100					
Juanito	0.66	10	90				
Marco	0.07	100					
Waty	0.42	66.66	16.67			16.67	
Nico	0.38						100
Victor	4.25		7.02	8.77	31.58	52.63	
Cheeta	4.76		100				
Coco	5.10		2		98		
Tom	0.39				20	80	

Wild‐born chimpanzees spent more scans displaying abnormal behavior (2.87 ± 2.40%) than their captive‐born counterparts (0.97 ± 1.62%); however, the difference was not significant (F_1, 4_ = 0.45, *p* = 0.54).

Former pet chimpanzees spent more scans displaying abnormal behavior (2.33% ± 2.18%) than entertainment chimpanzees (1.14% ± 2.03%); however, the difference was not significant (F_1, 4_ = 1.21, *p* = 0.33).

The LMM model showed no significant effect of age at rescue on the percentage of scans displaying abnormal behavior (F_1, 4_ = 6.77, *p* = 0.06).

Chimpanzees who grew up isolated spent more scans performing abnormal behavior (3.15% ± 2.17%) than chimpanzees previously housed with peers (0.32% ± 0.26%); however, the difference was not significant (F_1, 4_ = 1.12, *p* = 0.35). Table 2 summarizes the percentage of abnormal behaviors displayed by each individual.

### 3.2 Social Housing Can Mitigate the Percentage of Scans Spent Displaying Abnormal Behavior in Older Rescued Animals

**Figure 2:**
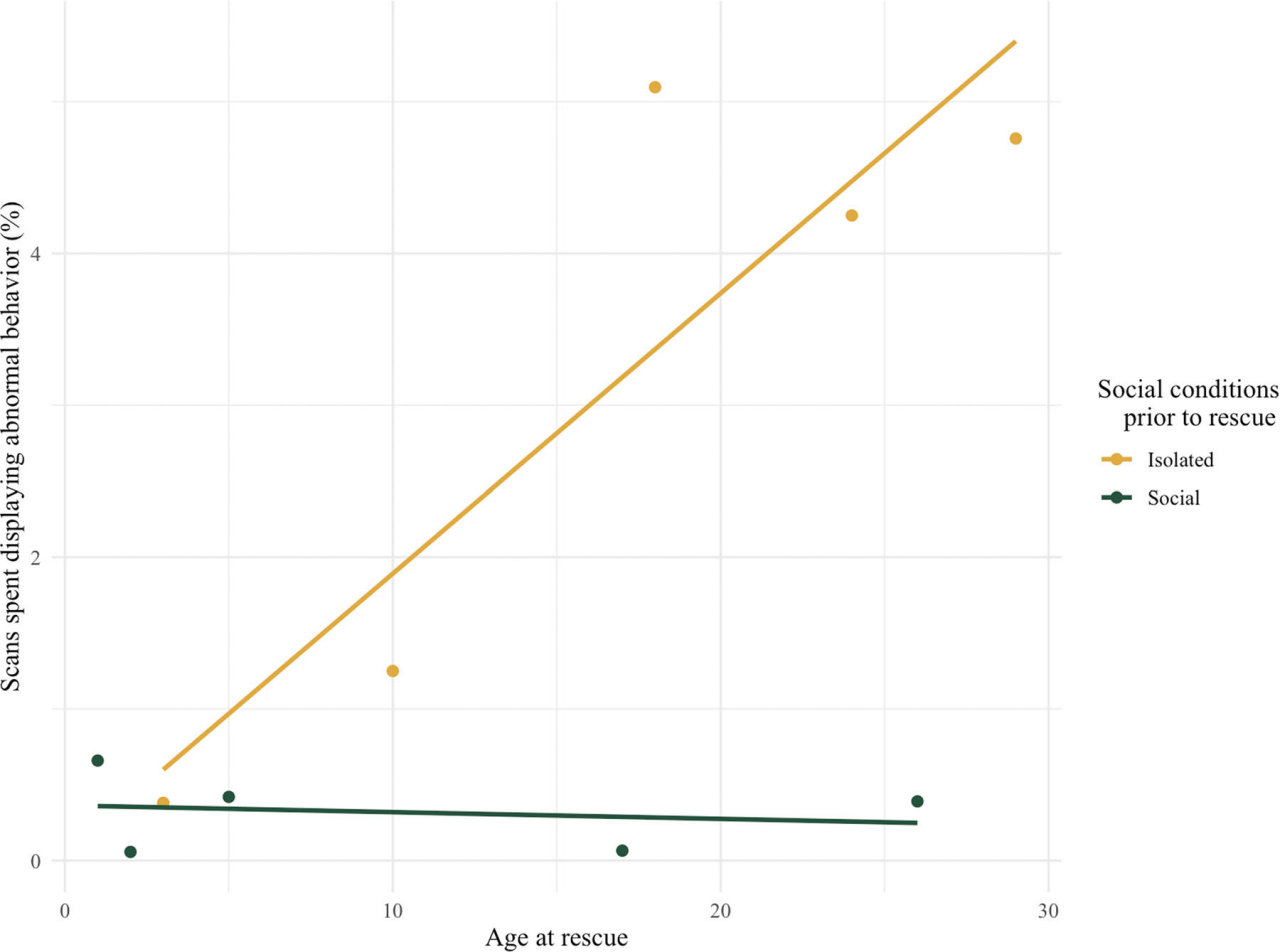
Relationship between abnormal behavior and age at rescue among chimpanzees with prior social experience (isolation or social housing).

The analysis of the interaction effect of the age at rescue and the chimpanzees' previous social conditions revealed that for individuals who grew up surrounded by peers, the percentage of scans spent displaying abnormal behavior did not differ based on the age at which they were rescued (slope = −0.004, std error: 0.01), whereas chimpanzees who were isolated beforehand showed an increase in the percentage of scans spent displaying abnormal behavior with the increase of age at rescue (slope = 0.18, std error: 0.05). The model revealed a statistically significant difference (F_1, 4_ = 11.98, *p* = 0.03), and the residuals were normally distributed (W = 0.90, *p* = 0.23) (Figure 2). The full model output is presented in Table S2.

## 4 Discussion

We explored the connection between individual histories of chimpanzees at the MONA sanctuary and the manifestation of abnormal behaviors during the study period. Our results showed that social isolation before rescue significantly increased the percentage of scans spent displaying abnormal behavior in chimpanzees rescued later in life. Moreover, this effect could be almost entirely mitigated if these animals were held in social groups before their rescue (Figure 2). This aligns with existing research suggesting that prolonged exposure to trauma has lasting effects on chimpanzee behavior (Kalcher et al. 2008; Kalcher‐Sommersguter et al. 2013), which have been compared with complex posttraumatic stress disorder (C‐PTSD) in humans with behaviors such as social withdrawal and self‐harm (Ferdowsian et al. 2011; Herman 1992; Dyer et al. 2009; Lopresti‐Goodman, Kameka, and Dube 2012; Bradshaw et al. 2008; Cantor and Price 2007). The implications of this finding reinforce the well‐established importance of socialization during infancy (Lopresti‐Goodman, Kameka, and Dube 2012; Crailsheim et al. 2020; Kalcher‐Sommersguter et al. 2015; Snyder et al. 1984) and adolescence (Reddy, Sandel, and Dahl 2022) for animal psycho‐social development.

Our study did not find differences in abnormal behavior among wild‐ or captive‐born individuals. This partly contradicts prior expectations that the higher level of social capacities shown by captive chimpanzees may correlate with less time spent displaying abnormal behavior and highlights the nuanced nature of individual experiences. One potential reason for the lack of observed differences is that we did not have information on the age at which the wild‐born chimpanzees were captured and placed in captivity, which may be a critical factor in understanding behavioral differences between these groups. Likewise, our data showed no differences between abnormal behaviors displayed by pet versus entertainment chimpanzees. A possible explanation arises from the labeling of individuals based on their primary roles as either pets or entertainers, whereas the practical reality is that most animals were involved in both capacities to some degree, complicating the clear differentiation of the influence of the two categories. Alternatively, the lack of observed differences between pet and entertainment chimpanzees may be attributed to both conditions being equally detrimental, leading to uniformly poor outcomes. While some of our comparisons did not reach statistical significance, certain trends may still be biologically meaningful. Notably, previously isolated chimpanzees displayed abnormal behaviors approximately 10 times more frequently than their previously peer‐housed counterparts. This difference, although not statistically significant, could reflect the long‐term impact of early‐life social deprivation. These results underscore the complexity of factors contributing to abnormal behavior (Birkett and Newton‐Fisher 2011; Nash et al. 1999; Goodall 1986; Whiten et al. 1999; Hook et al. 2002) and highlight the need for individualized care.

Moreover, it is important to mention that the levels of abnormal behavior recorded for 6 out of the 10 subjects in this study were notably low, with abnormal behaviors observed in fewer than 1% of scans for these individuals. This rate is lower than that reported for other captive chimpanzee population; for example, Wobber and Hare (2011) observed coprophagy and rocking in more than 6% and 4% of the total scans, respectively, in a group of 14 chimpanzees housed in at the Leipzig Zoo.

Overall, our findings provide valuable insights into the intricate relationship between individual life histories and abnormal behavior in chimpanzees. Future research addressing the effectiveness of different enrichment strategies in mitigating abnormal behavior could enhance our understanding and contribute to the well‐being of chimpanzees in sanctuary and zoo settings (Llorente et al. 2015; Feliu et al. 2022; van Leeuwen, Bruinstroop, and Haun 2022; Reimers, Schwarzenberger, and Preuschoft 2007; Kranendonk and Schippers 2014). Enrichment has been shown to enhance species‐typical behaviors, reduce agonism and stress‐related behaviors (Padrell et al. 2021; Bloomsmith and Lambeth 2000; Pomerantz and Terkel 2009; Yamanashi et al. 2016), whereas other studies did not find significant differences in behaviors correlated to enrichment, and highlighted the variation among individuals (Olsen et al. 2020; Greeson et al. 2022).

It is important to interpret our results with caution, because we had a limited sample size of 10 individuals equally divided into two groups. We also acknowledge both limited generalizability of the findings to other captive populations and observer bias as potential limitations of the study. Although observer bias is an inherent risk in observational research, at MONA we implemented a three‐step quality control and interobserver reliability testing protocol to reduce to the minimum the influence of observer bias. These factors should be considered when evaluating our conclusions, and future studies should aim to address these limitations by incorporating larger, more diverse samples. Our findings are consistent with existing literature on the matter, but more data are necessary for conclusive inferences about the relationship between abnormal behavior, age at rescue, and sociality before rescue for other chimpanzee groups as well as other primates.

## 5 Conclusion

Our findings prompt a broader consideration of the implications for primate welfare, urging a comprehensive approach to rehabilitation and care practices in zoos and sanctuaries. Caregivers need to learn to understand the effect of experiences pre‐rescue on the individuals' well‐being, and how to possibly reduce such impact. For instance, they could pay more attention to the needs of individuals rescued later in life and isolated before rescue, as these chimpanzees may exhibit higher levels of abnormal behavior and face greater challenges in social integration. Additionally, monitoring and supporting their mental health through behavioral assessments and enrichment strategies could help mitigate the effects of previous inadequate treatment and improve their overall quality of life.

## Author Contributions

**Emma Chen:** conceptualization (lead), data curation (lead), formal analysis (lead), investigation (equal), methodology (lead), writing–original draft (lead), writing–review and editing (lead). **Giulia Pipolo:** formal analysis (supporting), visualization (supporting), writing–review & editing (supporting). **Dietmar Crailsheim:** data curation (equal), methodology (equal), supervision (equal), writing–review and editing (equal). **Juliano Morimoto:** formal analysis (supporting), project administration (equal), supervision (equal), writing–review & editing (equal).

## Ethics Statement

The collection of data was based purely on behavioral observations and was conducted in accordance with all national and institutional guidelines for the care and management of primates as established by Fundación MONA, the Association for the Study of Animal Behavior/Animal Behavior Society and the Spanish Government (RD 53/2013).

## Conflicts of Interest

The authors declare no conflicts of interest.

## Supporting information

Supporting information.

## Data Availability

The data that support the findings of this study are available from the corresponding author upon reasonable request.

## References

[ajp23715-bib-0001] Altmann, J. 1974. “Observational Study of Behavior: Sampling Methods.” Behaviour 49, no. 3/4: 227–266. 10.1163/156853974x00534. 4597405

[ajp23715-bib-0002] Arcus Foundation . 2021. The Impact of Killing, Capture and Trade on Apes and Their Habitat. Killing, Capture, Trade and Ape Conservation State of the Apes, 23–47. Cambridge: Cambridge University Press. 10.1017/9781108768351.002.

[ajp23715-bib-0003] Ayuso, P. R. , O. Feliu , D. Riba , and D. Crailsheim . 2023. “Listening to Their Nights: Sleep Disruptions in Captive Housed Chimpanzees Affect Their Daytime Behavior.” Animals: An Open Access Journal From MDPI 13, no. 4: 696–696. 10.3390/ani13040696. 36830481 PMC9952389

[ajp23715-bib-0004] Baker, K. 2004. “Benefits of Positive Human Interaction for Socially‐Housed Chimpanzees.” Animal Welfare 13, no. 2: 239–245. 20505791 PMC2875797

[ajp23715-bib-0005] Baker, K. C. 2000. “Advanced Age Influences Chimpanzee Behavior in Small Social Groups.” Zoo Biology 19, no. 2: 111–119. 10.1002/1098-2361(2000)19:2<111::aid-zoo2>3.0.co;2-5.

[ajp23715-bib-0006] Baker, K. C. , and S. P. Easley . 1996. “An Analysis of Regurgitation and Reingestion in Captive Chimpanzees.” Applied Animal Behaviour Science 49, no. 4: 403–415. 10.1016/0168-1591(96)01061-1.

[ajp23715-bib-0007] Bates, D. , M. Mächler , B. Bolker , and S. Walker . 2015. “Fitting Linear Mixed‐Effects Models Using lme4.” Journal of Statistical Software 67, no. 1: 1–48. 10.18637/jss.v067.i01.

[ajp23715-bib-0008] Birkett, L. P. , and N. E. Newton‐Fisher . 2011. “How Abnormal Is the Behaviour of Captive, Zoo‐Living Chimpanzees?” PLoS One 6, no. 6: e20101. 10.1371/journal.pone.0020101. 21698219 PMC3116814

[ajp23715-bib-0009] Bloomsmith, M. A. , K. C. Baker , S. R. Ross , and S. P. Lambeth . 2006. “Early Rearing Conditions and Captive Chimpanzee Behavior: Some Surprising Findings.” In Nursery Rearing of Nonhuman Primates in the 21st Century. Developments in Primatology: Progress and Prospects, edited by G. P. Sackett , G. C. Ruppentahal , and K. Elias . Boston, MA: Springer. 10.1007/978-0-387-25640-5_15.

[ajp23715-bib-0010] Bloomsmith, M. A. , and S. P. Lambeth . 2000. “Videotapes as Enrichment for Captive Chimpanzees (*Pan troglodytes*).” Zoo Biology 19, no. 6: 541–551. 10.1002/1098-2361(2000)19:6<541::AID-ZOO6>3.0.CO;2-3. 11180415

[ajp23715-bib-0011] Botero, M. , S. E. MacDonald , and R. S. Miller . 2012. “Anxiety‐Related Behavior of Orphan Chimpanzees (*Pan troglodytes* Schweinfurthii) at Gombe National Park, Tanzania.” Primates 54, no. 1: 21–26. 10.1007/s10329-012-0327-1. 22976111

[ajp23715-bib-0012] Bradshaw, G. A. , T. Capaldo , L. Lindner , and G. Grow . 2008. “Building an Inner Sanctuary: Complex PTSD in Chimpanzees.” Journal of Trauma & Dissociation: The Official Journal of the International Society for the Study Of Dissociation (ISSD) 9, no. 1: 9–34. 10.1080/15299730802073619. 19042307

[ajp23715-bib-0013] Brüne, M. , U. Brüne‐Cohrs , W. C. McGrew , and S. Preuschoft . 2006. “Psychopathology in Great Apes: Concepts, Treatment Options and Possible Homologies to Human Psychiatric Disorders.” Neuroscience and Biobehavioral Reviews 30, no. 8: 1246–1259. 10.1016/j.neubiorev.2006.09.002. 17141312

[ajp23715-bib-0014] Cantor, C. , and J. Price . 2007. “Traumatic Entrapment, Appeasement and Complex Post‐Traumatic Stress Disorder: Evolutionary Perspectives of Hostage Reactions, Domestic Abuse and the Stockholm Syndrome.” Australian & New Zealand Journal of Psychiatry 41, no. 5: 377–384. 10.1080/00048670701261178. 17464728

[ajp23715-bib-0016] Cinini, S. M. , G. F. Barnabe , N. Galvã£o‐Coelho , et al. 2014. “Social Isolation Disrupts Hippocampal Neurogenesis in Young Non‐Human Primates.” Frontiers in Neuroscience 8: 8. 10.3389/fnins.2014.00045. 24733997 PMC3973924

[ajp23715-bib-0017] Crailsheim, D. , H. P. Stüger , E. Kalcher‐Sommersguter , and M. Llorente . 2020. “Early Life Experience and Alterations of Group Composition Shape the Social Grooming Networks of Former Pet and Entertainment Chimpanzees (*Pan troglodytes*).” PLoS One 15, no. 1: e0226947. 10.1371/journal.pone.0226947. 31940322 PMC6961849

[ajp23715-bib-0018] Davenport, R. K. 1963. “Stereotyped Behavior of the Infant Chimpanzee.” Archives of General Psychiatry 8, no. 1: 99–104. 10.1001/archpsyc.1963.01720070101013. 14025276

[ajp23715-bib-0019] Davenport, R. K. , C. M. Rogers , and D. M. Rumbaugh . 1973. “Long‐Term Cognitive Deficits in Chimpanzees Associated With Early Impoverished Rearing.” Developmental Psychology 9, no. 3: 343–347. 10.1037/h0034877.

[ajp23715-bib-0021] Dyer, K. F. W. , M. J. Dorahy , G. Hamilton , et al. 2009. “Anger, Aggression, and Self‐Harm in PTSD and Complex PTSD.” Journal of Clinical Psychology 65, no. 10: 1099–1114. 10.1002/jclp.20619. 19676110

[ajp23715-bib-0022] EARS . European Alliance for Rescue Centres and Sanctuaries: The Role of Rescue Centres and Sanctuaries. Available at. https://ears.org/ [Accessed 14 Dec. 2024].

[ajp23715-bib-0023] Eurogroup for Animals . 2021. *Wild Animals in EU Circuses*. Available at. https://www.eurogroupforanimals.org/files/eurogroupforanimals/2021-08/E4A-Circus_Report-09-08-2021.pdf.

[ajp23715-bib-0024] Feliu, O. , M. Masip , C. Maté , S. Sánchez‐López , D. Crailsheim , and E. Kalcher‐Sommersguter . 2022. “Behavioural Development of Three Former Pet Chimpanzees a Decade After Arrival at the MONA Sanctuary.” Animals: An Open Access Journal From MDPI 12, no. 2: 138. 10.3390/ani12020138. 35049762 PMC8772579

[ajp23715-bib-0025] Ferdowsian, H. R. , D. L. Durham , C. Kimwele , et al. 2011. “Signs of Mood and Anxiety Disorders in Chimpanzees.” PLoS One 6, no. 6: e19855. 10.1371/journal.pone.0019855. 21698223 PMC3116818

[ajp23715-bib-0026] Fox, J. , and S. Weisberg . 2019. An R Companion to Applied Regression Third edition. Thousand Oaks, CA: Sage. https://socialsciences.mcmaster.ca/jfox/Books/Companion/.

[ajp23715-bib-0027] Freeman, H. D. , and S. R. Ross . 2014. “The Impact of Atypical Early Histories on Pet or Performer Chimpanzees.” PeerJ (Corta Madera, CA and London) 2: e579. 10.7717/peerj.579. PMC4179557 25279262

[ajp23715-bib-0028] Fundaciónmona.org . 2023. *The Center | Fundación Mona*. [online] Available at. https://fundacionmona.org/en/el-centre/.

[ajp23715-bib-0029] Goodall, J. 1983. “Population Dynamics During a 15 Year Period in One Community of Free‐Living Chimpanzees in the Gombe National Park, Tanzania.” Zeitschrift für Tierpsychologie 61, no. 1: 1–60. 10.1111/j.1439-0310.1983.tb01324.x.

[ajp23715-bib-0030] Goodall, J. 1986. The Chimpanzees of Gombe: Patterns of Behavior. Cambridge, MA: Harvard University Press.

[ajp23715-bib-0031] Greeson, J. L. , K. I. Gabriel , J. B. Mulcahy , B. King Hendrickson , S. D. Lonborg , and J. C. Holloway . 2022. “An Evaluation of Ethograms Measuring Distinct Features of Enrichment Use by Captive Chimpanzees (*Pan troglodytes*).” Animals: An Open Access Journal From MDPI 12, no. 16: 2029. 10.3390/ani12162029. 36009618 PMC9404423

[ajp23715-bib-0032] Herman, J. L. 1992. “Complex PTSD: A Syndrome in Survivors of Prolonged and Repeated Trauma.” Journal of Traumatic Stress 5: 377–391. 10.1002/jts.2490050305.

[ajp23715-bib-0033] Hook, M. A. , S. P. Lambeth , J. E. Perlman , R. Stavisky , M. A. Bloomsmith , and S. J. Schapiro . 2002. “Inter‐Group Variation in Abnormal Behavior in Chimpanzees (*Pan troglodytes*) and Rhesus Macaques (*Macaca Mulatta*).” Applied Animal Behaviour Science 76, no. 2: 165–176. 10.1016/s0168-1591(02)00005-9.

[ajp23715-bib-0034] van Ijzendoorn, M. H. , K. A. Bard , M. J. Bakermans‐Kranenburg , and K. Ivan . 2009. “Enhancement of Attachment and Cognitive Development of Young Nursery‐Reared Chimpanzees in Responsive Versus Standard Care.” Developmental Psychobiology 51, no. 2: 173–185. 10.1002/dev.20356. 19016474

[ajp23715-bib-0035] Iossa, G. , C. Soulsbury , and S. Harris . 2009. “Are Wild Animals Suited to a Travelling Circus Life?” Animal Welfare 18, no. 2: 129–140. 10.1017/S0962728600000270.

[ajp23715-bib-0036] Jacobson, S. L. , H. D. Freeman , R. M. Santymire , and S. R. Ross . 2017. “Atypical Experiences of Captive Chimpanzees (*Pan troglodytes*) Are Associated With Higher Hair Cortisol Concentrations as Adults.” Royal Society Open Science 4, no. 12: 170932. 10.1098/rsos.170932. 29308234 PMC5750001

[ajp23715-bib-0037] Kalcher, E. , C. Franz , K. Crailsheim , and S. Preuschoft . 2008. “Differential Onset of Infantile Deprivation Produces Distinctive Long‐Term Effects in Adult Ex‐Laboratory Chimpanzees (*Pan troglodytes*).” Developmental Psychobiology 50, no. 8: 777–788. 10.1002/dev.20330. 18688804

[ajp23715-bib-0038] Kalcher‐Sommersguter, E. , C. Franz‐Schaider , S. Preuschoft , and K. Crailsheim . 2013. “Long‐Term Evaluation of Abnormal Behavior in Adult Ex‐Laboratory Chimpanzees (*Pan troglodytes*) Following Re‐Socialization.” Behavioral Sciences 3, no. 1: 99–119. 10.3390/bs3010099. 25379228 PMC4217617

[ajp23715-bib-0039] Kalcher‐Sommersguter, E. , S. Preuschoft , C. Franz‐Schaider , C. K. Hemelrijk , K. Crailsheim , and J. J. M. Massen . 2015. “Early Maternal Loss Affects Social Integration of Chimpanzees Throughout Their Lifetime.” Scientific Reports 5, no. 1: 16439. 10.1038/srep16439. 26552576 PMC4639738

[ajp23715-bib-0040] Kawanaka, K. 1989. “Age Differences in Social Interactions of Young Males in a Chimpanzee Unit‐Group at the Mahale Mountains National Park, Tanzania.” Primates 30, no. 3: 285–305. 10.1007/bf02381256.

[ajp23715-bib-0041] Kempes, M. M. , M. M. C. Gulickx , H. J. C. van Daalen , A. L. Louwerse , and E. H. M. Sterck . 2008. “Social Competence Is Reduced in Socially Deprived Rhesus Monkeys (*Macaca Mulatta*).” Journal of Comparative Psychology 122, no. 1: 62–67. 10.1037/0735-7036.122.1.62. 18298282

[ajp23715-bib-0042] Kranendonk, G. , and E. P. Schippers . 2014. “A Pilot Study on the Effects of a Change in Behavioural Management on the Behaviour of Captive Chimpanzees (*Pan troglodytes*).” Applied Animal Behaviour Science 160: 127–137. 10.1016/j.applanim.2014.09.008.

[ajp23715-bib-0043] Kühl, H. S. , C. Boesch , L. Kulik , et al. 2019. “Human Impact Erodes Chimpanzee Behavioral Diversity.” Science 363, no. 6434: 1453–1455. 10.1126/science.aau4532. 30846610

[ajp23715-bib-0044] Kuznetsova, A. , P. B. Brockhoff , and R. H. B. Christensen . 2017. “lmerTest Package: Tests in Linear Mixed Effects Models.” Journal of Statistical Software 82, no. 13: 1–26. 10.18637/jss.v082.i13.

[ajp23715-bib-0045] van Leeuwen, E. J. C. , B. M. C. Bruinstroop , and D. B. M. Haun . 2022. “Early Trauma Leaves No Social Signature in Sanctuary‐Housed Chimpanzees (*Pan troglodytes*).” Animals: An Open Access Journal From MDPI 13, no. 1: 49. 10.3390/ani13010049. 36611659 PMC9817851

[ajp23715-bib-0046] Llorente, M. , D. Riba , S. Ballesta , O. Feliu , and C. Rostán . 2015. “Rehabilitation and Socialization of Chimpanzees (*Pan troglodytes*) Used for Entertainment and as Pets: An 8‐Year Study at Fundació Mona.” International Journal of Primatology 36, no. 3: 605–624. 10.1007/s10764-015-9842-4.

[ajp23715-bib-0047] López‐Álvarez, J. , Y. Sanjorge , S. Soloaga , D. Crailsheim , and M. Llorente . 2019. “Looking for Visitor's Effect in Sanctuaries: Implications of Guided Visitor Groups on the Behavior of the Chimpanzees at Fundació Mona.” Animals: An Open Access Journal From MDPI 9, no. 6: 347. 10.3390/ani9060347. 31200436 PMC6617045

[ajp23715-bib-0048] Lopresti‐Goodman, S. , M. Kameka , and A. Dube . 2012. “Stereotypical Behaviors in Chimpanzees Rescued From the African Bushmeat and Pet Trade.” Behavioral Sciences 3, no. 1: 1–20. 10.3390/bs3010001. 25379223 PMC4217614

[ajp23715-bib-0049] Lutz, C. K. , K. Coleman , L. M. Hopper , M. A. Novak , J. E. Perlman , and O. Pomerantz . 2022. “Nonhuman Primate Abnormal Behavior: Etiology, Assessment, and Treatment.” American Journal of Primatology 84, no. 6: e23380. 10.1002/ajp.23380. 35383995 PMC9586202

[ajp23715-bib-0050] Maki, S. , J. Fritz , and N. England . 1993. “An Assessment of Early Differential Rearing Conditions on Later Behavioral Development in Captive Chimpanzees.” Infant Behaviour & Development 16, no. 3: 373–381. 10.1016/0163-6383(93)80042-7.

[ajp23715-bib-0051] Mason, G. 2006. “Stereotypic Behaviour in Captive Animals: Fundamentals and Implications for Welfare and Beyond.” In Stereotypic Animal Behaviour: Fundamentals and Applications to Welfare, edited by G. Mason and J. Rushen , 2nd ed., 325–351. Oxfordshire, UK: CABI eBooks. 10.1079/9780851990040.0000.

[ajp23715-bib-0052] Menzel Jr., E. W. , R. K. Davenport Jr. , and C. M. Rogers . 1963. “Effects of Environmental Restriction Upon the Chimpanzee's Responsiveness in Novel Situations.” Journal of Comparative and Physiological Psychology 56, no. 2: 329–334. 10.1037/h0038718. 13934894

[ajp23715-bib-0054] Nash, L. T. , J. Fritz , P. A. Alford , and L. Brent . 1999. “Variables Influencing the Origins of Diverse Abnormal Behaviors in a Large Sample of Captive Chimpanzees (*Pan troglodytes*).” American Journal of Primatology 48, no. 1: 15–29. 10.1002/(sici)1098-2345(1999)48:1<15::aid-ajp2>3.0.co;2-r. 10326768

[ajp23715-bib-0055] Neal, S. J. , J. Hau , S. P. Lambeth , and S. J. Schapiro . 2019b. “Differences in Behaviour Between Elderly and Nonelderly Captive Chimpanzees and the Effects of the Social Environment.” Journal of the American Association for Laboratory Animal Science 58, no. 6: 783–789. 10.30802/aalas-jaalas-19-000019. 31645233 PMC6926406

[ajp23715-bib-0056] Neal Webb, S. J. , J. Hau , and S. J. Schapiro . 2019a. “Does Group Size Matter? Captive Chimpanzee (*Pan troglodytes*) Behavior as a Function of Group Size and Composition.” American Journal of Primatology 81, no. 1: e22947. 10.1002/ajp.22947. 30620093 PMC6472487

[ajp23715-bib-0057] Olsen, A. M. , H. G. Kristensen , K. W. Iversen , et al. 2020. “Assessment of Abnormal Behaviour and the Effect of Enrichment on Captive Chimpanzees in Aalborg Zoo.” GABJ 4, no. 2: 73–91. 10.46325/gabj.v4i2.99.

[ajp23715-bib-0058] Ongman, L. , C. Colin , E. Raballand , and T. Humle . 2013. “The ‘Super Chimpanzee’: The Ecological Dimensions of Rehabilitation of Orphan Chimpanzees in Guinea, West Africa.” Animals: An Open Access Journal From MDPI 3, no. 1: 109–126. 10.3390/ani3010109. 26487312 PMC4495513

[ajp23715-bib-0059] Ortín, S. , Y. Úbeda , R. M. Garriga , and M. Llorente . 2019. “Bushmeat Trade Consequences Predict Higher Anxiety, Restraint, and Dominance in Chimpanzees.” Developmental Psychobiology 61, no. 6: 874–887. 10.1002/dev.21853. 30957221

[ajp23715-bib-0060] Padrell, M. , F. Amici , M. P. Córdoba , et al. 2021. “Artificial Termite‐Fishing Tasks as Enrichment for Sanctuary‐Housed Chimpanzees: Behavioral Effects and Impact on Welfare.” Animals: An Open Access Journal From MDPI 11, no. 10: 2941. 10.3390/ani11102941. 34679962 PMC8532803

[ajp23715-bib-0061] Padrell, M. , D. Riba , Y. Úbeda , F. Amici , and M. Llorente . 2020. “Personality, Cognition and Behavior in Chimpanzees: A New Approach Based on Eysenck's Model.” PeerJ 8: e9707. 10.7717/peerj.9707. 32874782 PMC7439959

[ajp23715-bib-0062] Pomerantz, O. , and J. Terkel . 2009. “Effects of Positive Reinforcement Training Techniques on the Psychological Welfare of Zoo‐Housed Chimpanzees (*Pan troglodytes*).” American Journal of Primatology 71, no. 8: 687–695. 10.1002/ajp.20703. 19434627

[ajp23715-bib-0063] R Core Team . 2018. R: A Language and Environment for Statistical Computing. Vienna, Austria: R Foundation for Statistical Computing. https://www.R-project.org/.

[ajp23715-bib-0064] Reddy, R. B. , A. A. Sandel , and R. E. Dahl . 2022. “Puberty Initiates a Unique Stage of Social Learning and Development Prior to Adulthood: Insights From Studies of Adolescence in Wild Chimpanzees.” Developmental Cognitive Neuroscience 58: 101176. 10.1016/j.dcn.2022.101176. 36427434 PMC9699942

[ajp23715-bib-0065] Reimers, M. , F. Schwarzenberger , and S. Preuschoft . 2007. “Rehabilitation of Research Chimpanzees: Stress and Coping After Long‐Term Isolation.” Hormones and Behavior 51, no. 3: 428–435. 10.1016/j.yhbeh.2006.12.011. 17292368

[ajp23715-bib-0066] Ross, M. R. , T. Niemann , J. D. Wark , et al. (2016). *ZooMonitor* (Version 1) [Mobile Application Software]. https://zoomonitor.org.

[ajp23715-bib-0067] Sandel, A. A. , K. E. Langergraber , and J. C. Mitani . 2020. “Adolescent Male Chimpanzees (*Pan troglodytes*) Form Social Bonds With Their Brothers and Others During the Transition to Adulthood.” American Journal of Primatology 82, no. 1: e23091. 10.1002/ajp.23091. 31903634 PMC7015449

[ajp23715-bib-0068] Sandel, A. A. , R. B. Reddy , and J. C. Mitani . 2017. “Adolescent Male Chimpanzees Do Not Form a Dominance Hierarchy With Their Peers.” Primates 58: 39–49. 10.1007/s10329-016-0553-z. 27379650 PMC5450613

[ajp23715-bib-0069] Snyder, D. S. , C. E. Graham , J. A. Bowen , and M. Reite . 1984. “Peer Separation in Infant Chimpanzees, a Pilot Study.” Primates 25, no. 1: 78–88. 10.1007/bf0238229.

[ajp23715-bib-0070] Suomi, S. J. , H. F. Harlow , and S. D. Kimball . 1971. “Behavioral Effects of Prolonged Partial Social Isolation in the Rhesus Monkey.” Psychological Reports 29, no. 3_suppl: 1171–1177. 5003142 10.2466/pr0.1971.29.3f.1171

[ajp23715-bib-0071] Turner, C. H. , R. K. Davenport Jr. , and C. M. Rogers . 1969. “The Effect of Early Deprivation on the Social Behavior of Adolescent Chimpanzees.” American Journal of Psychiatry 125, no. 11: 1531–1536. 10.1176/ajp.125.11.1531. 5776861

[ajp23715-bib-0072] Veenema, H. C. , B. M. Spruijt , W. H. Gispen , and J. A. R. A. M. van Hooff . 1997. “Aging, Dominance History, and Social Behavior in Java‐Monkeys (*Macaca Fascicularis*).” Neurobiology of Aging 18, no. 5: 509–515. 10.1016/s0197-4580(97)00107-3. 9390777

[ajp23715-bib-0073] Whiten, A. , J. Goodall , W. C. McGrew , et al. 1999. “Cultures in Chimpanzees.” Nature 399, no. 6737: 682–685. 10.1038/21415. 10385119

[ajp23715-bib-0074] Wickham, H. 2016. ggplot2: Elegant Graphics for Data Analysis, 11–31. New York, NY: Springer‐Verlag.

[ajp23715-bib-0075] Wickham, H. , R. François , L. Henry , K. Müller , and D. Vaughan . 2023. *dplyr: A Grammar of Data Manipulation*. R package version 1.1.0. https://CRAN.R-project.org/package=dplyr.

[ajp23715-bib-0076] Wobber, V. , and B. Hare . 2011. “Psychological Health of Orphan Bonobos and Chimpanzees in African Sanctuaries.” PLoS One 6, no. 6: e17147. 10.1371/journal.pone.0017147. 21666743 PMC3110182

[ajp23715-bib-0077] Yamanashi, Y. , M. Matsunaga , K. Shimada , R. Kado , and M. Tanaka . 2016. “Introducing Tool‐Based Feeders to Zoo‐Housed Chimpanzees as a Cognitive Challenge: Spontaneous Acquisition of New Types of Tool Use and Effects on Behaviors and Use of Space.” Journal of Zoo and Aquarium Research 4, no. 3: 147–155. 10.19227/jzar.v4i3.235.

