# Supplementary material for: The Lasting Impact of Social Isolation: Behavioral Insights From Former Pet and Entertainer Chimpanzees in a Sanctuary in Spain
Source: Am J Primatol. 2024 Dec 18;87(1):e23715. doi: 10.1002/ajp.23715 (PMC11655702; doi:10.1002/ajp.23715)
Supplement: Supplementary file 1 — Supporting information. [file AJP-87-e23715-s001.docx]

**Supplementary material**

**The lasting impact of social isolation: behavioral insights from former pet and entertainer chimpanzees in a sanctuary in Spain.**

Authorship list: Emma Chen^1,*^, Giulia Pipolo^1^, Dietmar Crailsheim^2^, Juliano Morimoto^3,4^

**
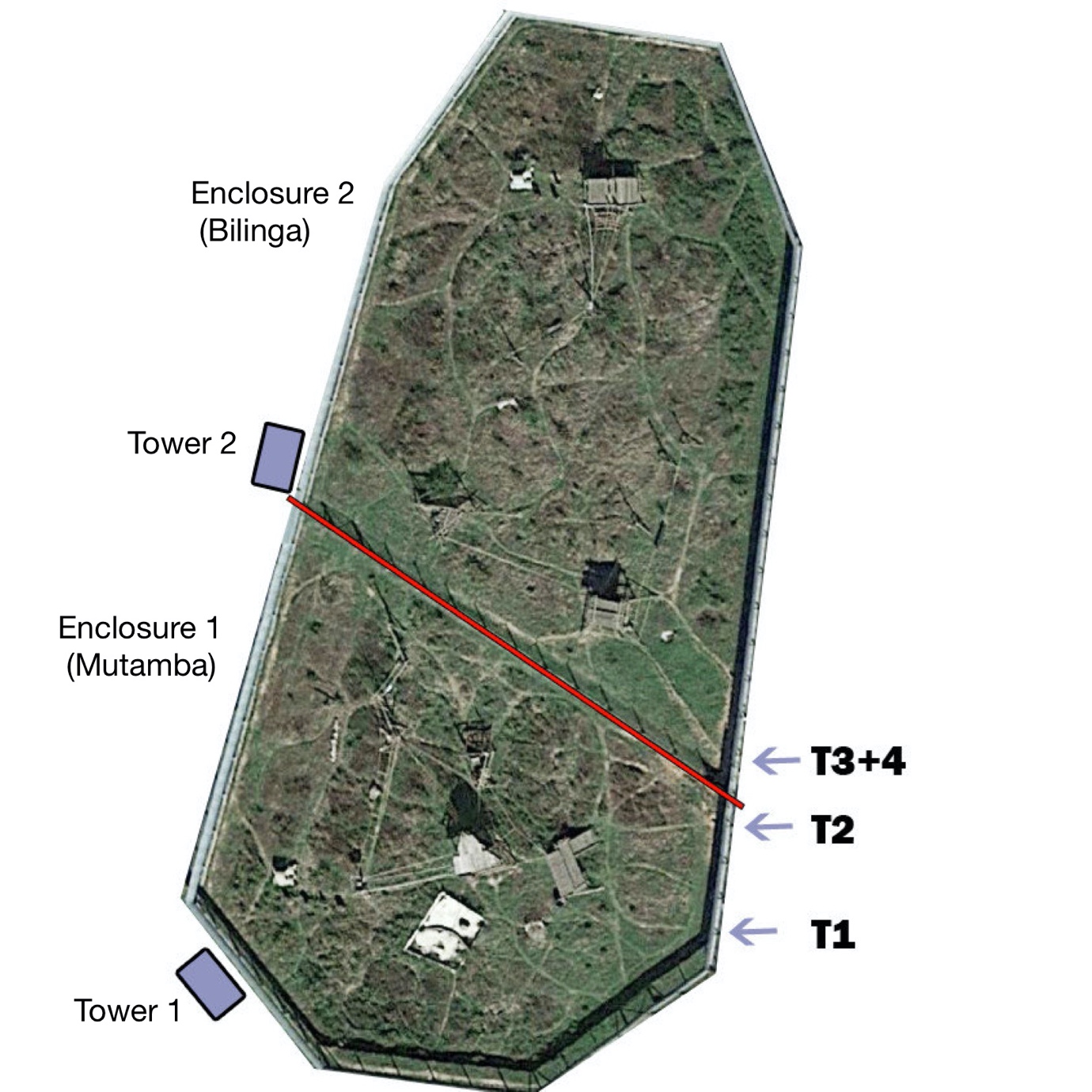
**

**Figure S1***.* Aerial view of the two enclosures for the focal groups of chimpanzees, *Mutamba* and *Bilinga*, housed at Fundación MONA. Towers 1 and 2 are the viewpoints from where the observational sessions are conducted. T1, T2, T3 and T4 are the entrances to the indoor enclosures. Map provided by Fundación MONA.

**Table S1.** Full ethogram illustrating all the behaviors observed in the chimpanzee groups at Fundación MONA, including the different types of abnormal behaviors (included in the solitary behavior channel) identified in the groups considered in this study.

|  |  |  |  |  |
| --- | --- | --- | --- | --- |
| **Interval: SOLITARY BEHAVIOR CHANNEL** | | | | |
| Inactivity | Resting |  | Stationary without actively interacting with himself, others or the environment. | |
|  | Vigilance |  | Fixed gaze at something or someone or scanning the distant environment. It includes looking at distant group members, other groups, and the surrounding area. Mark the target of the behavior: Own group, Other group, Visitors, Observers, Head keeper, Caregiver, Office staff, Veterinarian, Technician, Macaques. | |
|  | Relaxed resting |  | Dozing or sleeping in an upward-facing or lateral position with either elongated or bent limbs (without hugging oneself). The individual does not show any indication of being focused on himself, any other individual or their general environment. The individual is not exhibiting RELAXED RESTING if he goes into a fetal position (may include hugging limbs and/or patting oneself with fingers on various parts of the body), nor any other behavior categorized as abnormal, such as: self-poking or self-clasping, etc. | |
| Alimentation | Feeding |  | Ingest or handle solid or liquid food in a stationary position. | |
|  | Foraging |  | Active search and/or transport of food (the animal keeps its eyes focused on possible food sources). It can include punctual food intake without interrupting gathering food. | |
|  | Food extraction |  | The chimpanzee is working an enrichment device/item (provided by care givers) which contains food items. | |
| Locomotion | |  | Displacement from a point A to a point B on a vertical or horizontal surface, while not exhibiting any other behavior from the behavioral catalogue. | |
| Manipulation | |  | Inspecting elements of the environment or non-food enrichment with the upper or lower extremities. May include using the mouth, but not the mouth alone. | |
| Self-directed | |  | Behaviors directed towards the individual (own body), such as self-cleaning (auto grooming), self-inspection, masturbation, scratching, scrubbing, body inspection, yawning or manipulation of specific body parts. | |
| Solitary play | |  | Ludicrous behaviors exhibited while being alone. Has to be (at times) accompanied by either laughing vocalizations, a "play face", acrobatic body movements and/or galloping. May include the use of the environment or objects. | |
| Abnormal |  |  | Behaviors not typically seen in wild populations or normal behaviors exhibited in an excessive manner or as an inadequate, non-adaptive response to a specific situation: | |
|  | Self-scratch |  | Invariable, rhythmic and repetitive movement of the fingers or with an object (e.g. stick) rubbing some part of its own body. | |
|  | Self-poke |  | Inserting, pressing or twisting of fingers or objects (e.g. sticks) repeatedly or continuously in a part of the body (only mark when actively manipulating or maintaining contact). Typically ears, nose, eye socket, armpit or anal area. | |
|  | Self-clap |  | Hitting (soft or hard) a specific part of the body with hands or feet rhythmically and repetitively. | |
|  | Rocking |  | Repetitive and invariable swinging back and forth of the body while sitting (they can hug or grab objects at the same time, (e.g. pile of straw). | |
|  | Over grooming |  | Excessive self-cleaning behavior of the skin / coat with hands or mouth, which may include pulling and ingesting hair. The behavior can also be focused on wounds or damage to the skin, deteriorating its condition. They usually do it on the same areas of the body with a visible lack of hair. | |
|  | Coprophagy |  | Handling and/or ingesting of feces (both own and others). | |
|  | Lip picking |  | Localized lip chewing with canine and/or manipulation with fingers. May lead to bleeding. | |
|  | Touch forehead |  | Prolonged touch or manipulation of forehead occipital bone (area around eyes). | |
|  | Other abnormal |  | Other abnormal or stereotypical behaviors not described above. The majority can be identified due to its rhythmically, repetitiveness and/or excessive frequency. | |
| Other individual | |  | Behaviors exhibited while being alone that are not better defined by any other category of the "Solitary Behavior" group. | |
| Human interaction |  |  | Any behaviors directed or related to humans in proximity. | Mark the target of the behavior: Visitors, Observers, Head keeper, Caregiver, Office staff, Veterinarian, Technician. |
|  | Human display |  | Chimpanzees show threatening behavior and/or perform an agonistic display directed at humans in close proximity. It may include attempts to come into physical contact, throw items, or spit. |  |
|  | Human submissive |  | Chimpanzees are agitated with a scared face can include behaviors such as asking for support, crying or yelling directed at a human. |  |
|  | Human affiliative |  | Neutral or positive social interaction between the chimpanzee and a human. Includes calling the attention, greetings, as well as physical contact of non-agonistic nature. It can be initiated by animals or by humans. |  |
|  | Human follow |  | Synchronized locomotion in parallel to the fence following the path that people take around the enclosure. |  |
|  | Human observe close up |  | The individual remains less than 1.5 meters from the fence observing humans in close proximity without interacting in any way. |  |
| Not visible |  |  | There is no or insufficient information regarding the behavior the animal is exhibiting: | |
|  | Not located |  | Observer does not know/see where the chimpanzee is. | |
|  | Interior |  | Chimpanzee has access to both indoor and outdoor enclosures and is located in the not observable indoor facility. | |
|  | blurred solitary |  | The individual can be located and is clearly performing an individual behavior but the behavior cannot be identified due to lack of vision (e.g. being in the shade, showing the back or is partly behind a structure). | |
|  | blurred affiliative |  | The individual can be located and is clearly performing an affiliative behavior but the behavior cannot be identified due to lack of vision (e.g. being in the shade, showing the back or is partly behind a structure). | |
|  | blurred agonistic |  | The individual can be located and is clearly performing an agonistic behavior but the behavior cannot be identified due to lack of vision (e.g. being in the shade, showing the back or is partly behind a structure). | |
|  |  |  |  |  |
| **Interval: INTERACTIVE BEHAVIOR CHANNEL** | | | | |
| Grooming | |  | Body-cleansing behavior from one individual to another (includes mutual grooming), performed with the upper extremities or with the mouth. | |
| Affiliative |  |  | This includes all socio-positive behaviors directed and/or received by another member of the same group. | |
|  | Social Play |  | Ludicrous behavior between two or more individuals punctually accompanied by playful indicators (e.g., play face, laugh, gallop, cartwheels and pirouettes). Tag, catch and grab limbs or objects, tickles, muzzling, rough-and-tumble. These interactions do not require permanent physical contact. | |
|  | Follow |  | Synchronized movement of walking behind or beside another individual, following the trajectory of one or several chimpanzees (always mark mutual). Chimpanzee might touch in occasions, but might also maintain a big distance. | |
|  | Feed together |  | Two or more chimpanzee feed from the same food source or forage in close proximity, sharing/allowing the collection of food within their reach by another individual, sharing the food in a passive way (always mark mutual). | |
|  | Embrace |  | An individual hugs or tries to hug another with one or both arms in the absence of an agonistic event or agonistic indicators such as aggressive or submissive mimics. This behavior can occur while stationary or while in movement. | |
|  | Other affiliative |  | Other affiliative-like behaviors that cannot be defined by any of the listed social behaviors. | |
| Agonistic |  |  | This includes all aggressive or submissive behaviors directed and/or received by another member of the same group. | |
|  | Aggression |  | Behaviors relating to the agonistic/display threat, direct aggression, wounding, chasing, displacement and/or resource appropriation (including object and social resources). It can be accompanied by vocalizations. Chimpanzee is typically choosing more dominant and impressive body position to appear bigger and threatening. Movements are broad and often contain drumming, swaggering, stomping etc. | |
|  | Submission |  | Behaviors indicating fear or subordinate intentions toward one or several other chimpanzees. The chimpanzee shows signs of fear directed at another individual in an agonistic context. It may include approaching and presenting vulnerable body parts in an attempt to appease or attempt to avoid or flee from an individual displaying aggressive behavior. It can include actions such as genital presenting, extending limbs, hand to mouth, finger to mouth, running away, avoiding, and is typically accompanied by a scared face and vocalizations such as yelling, screaming, crying or pant-grunting. | |
|  | Other agonistic |  | Other aggression-related behaviors that cannot be defined by Aggression or Submission. | |
|  | Display undirected |  | Individual shows off strength, but towards no one in particular. May include vocalizations such as strong lip splutter, hooing, or hooting. Chimpanzee tends to be pilo erected, running, swaggering, stomping the ground, throwing objects or hitting elements or structures. | |
|  | Display other species |  | Individual shows off strength specifically towards an animal of a different species (e.g. Barbary macaques), often while approaching said animal(s) gradually. May include vocalizations such as strong lip splutter, hooing, or hooting. Chimpanzee tends to be pilo-erected, running, swaggering, stomping the ground, throwing objects or hitting elements or structures. | |
| Socio sexual | |  | Sexual interaction, or attempt of sexual interaction, between two individuals. Includes behaviors such as: copulation, attempted copulation, genital presentation, genital inspection and other behaviors directed towards the genital area of another individual. Choose between Genital inspection or copulation. In the latter the male chimpanzee would mount, including thrust movements, the female at one point (the mounting might only last a few seconds). Always mark mutual. | |
| Interaction with other group | |  | Any social interactions directed and/or received by members of a neighboring chimpanzee group. Social sub behaviors (i.e., Aggression, Display, Submission, Social play, Follow, Other affiliative, Other agonistic) are defined identically as in in-group interactions. Here Directionality has to be marked within the observed chimpanzee. | |

**Table S2.** Linear mixed model output based on ANOVA Type III Analysis. (*) indicates significant p value (<0.05).

| **ANOVA Type III Analysis of Variance Table with Satterthwaite's method** | | | | |
| --- | --- | --- | --- | --- |
|  | SumSq | MeanSq | Fvalue | p |
| Origin | 0.3412 | 0.3412 | 0.4549 | 0.53699 |
| Early_history | 0.9102 | 0.9102 | 1.2134 | 0.33249 |
| Age_at_rescue | 5.0804 | 5.0804 | 6.7725 | 0.05990 |
| Social_conditions | 0.8422 | 0.8422 | 1.1227 | 0.34908 |
| Age_at_rescue:Social_conditions | 8.9879 | 8.9879 | 11.9815 | 0.02578 (*) |
